# Association between Sugar-Sweetened Beverage Consumption and the Risk of the Metabolic Syndrome: A Systematic Review and Meta-Analysis

**DOI:** 10.3390/nu15020430

**Published:** 2023-01-13

**Authors:** Ainara Muñoz-Cabrejas, Pilar Guallar-Castillón, Martín Laclaustra, Helena Sandoval-Insausti, Belén Moreno-Franco

**Affiliations:** 1 Instituto de Investigación Sanitaria Aragón, Hospital Universitario Miguel Servet, 50009 Zaragoza, Spain; 2 Departamento de Medicina Preventiva y Salud Pública, Universidad Autónoma de Madrid-IdiPaz, CIBERESP (CIBER de Epidemiología y Salud Pública), IMDEA Alimentación, 28029 Madrid, Spain; 3 Departamento de Medicina, Psiquiatría y Dermatología, Universidad de Zaragoza, 50009 Zaragoza, Spain; 4 CIBERCV (CIBER de Enfermedades Cardiovasculares), 28029 Madrid, Spain; 5 Department of Nutrition, Harvard T.H. Chan School of Public Health, Boston, MA 02115, USA; 6 Departamento de Medicina, Preventiva y Salud Pública, Universidad de Zaragoza, 50009 Zaragoza, Spain

**Keywords:** sugar-sweetened beverages, metabolic syndrome, cardiovascular disease, systematic review, meta-analysis

## Abstract

(1) Background: The increasing occurrence of the metabolic syndrome (MetS) is largely related to harmful food habits. Among them, the consumption of sugar-sweetened beverages (SSBs) is noteworthy. However, to our knowledge, there are not enough high-quality methodological studies summarizing the association between the intake of SSBs and the MetS. Therefore, the aim of this study is to examine the existing published results on this association among adults by synthesizing the existing evidence. (2) Methods: Systematic review and meta-analysis of observational studies following the PRISMA guidelines. Relevant information was extracted and presented following the PRISMA recommendations. PubMed and SCOPUS databases were searched for studies published until June 2022 that assessed the association between SSB consumption (including soft drinks, bottled fruit juices, energy drinks, and milkshakes) and the occurrence of MetS. Random effect models were used to estimate pooled odds ratios (ORs) with their 95% coefficient interval, and I^2^ was used to assess heterogeneity. (3) Results: A total of 14 publications from 6 different countries were included in this meta-analysis (9 cross-sectional and 5 cohort studies). For the cross-sectional studies, which included 62,693 adults, the pooled OR for the risk of MetS was 1.35 (95% CI 1.15, 1.58; I^2^ 57%) when the highest versus the lowest categories of SSB consumption were compared. For the cohort studies, which included 28,932 adults, the pooled OR was 1.18 (95% CI 1.06, 1.32; I^2^ 70%). (4) Conclusions: The consumption of SSBs was positively associated with an increased risk of MetS. The published literature supports public health strategies and the need to reduce the consumption of SSBs to prevent MetS.

## 1. Introduction

The metabolic syndrome (MetS) is a cluster of cardiovascular risk factors that includes atherogenic dyslipidemia, abdominal obesity, high blood pressure, as well as high blood glucose. The MetS has been positively associated with the development of type 2 diabetes mellitus and cardiovascular disease (CVD) [1,2]. Thus, it has been showed that adults who have the MetS are at twice the risk of developing CVD over the next five-to-ten years when compared to adults without the MetS [3]. 

The high prevalence of MetS worldwide has turned it into a public health concern [4,5,6,7,8], varying from 12.5% to 31.4% according to the diagnosis definition used [7]. This increase is largely due to unhealthy eating habits among the population. Out of these unhealthy eating habits, the intake of added sugars is still exceeding the limits of 10% of the recommended daily calories [9,10]. There is a growing worry that the intake of added sugars derives in a positive energy balance, contributing to an increase in weight gain [9,10], obesity [10,11], type 2 diabetes [12,13], and finally an increased risk of developing the MetS and CVD [13,14]. 

Part of this intake of added sugars comes from sugar-sweetened beverage (SSB) consumption, which includes soft drinks, bottled fruit juices, energy drinks, as well as milkshakes. Artificially sweetened beverages (ASBs) are not considered to be SSBs, since sugar is not used within their manufacturing process.

The available evidence from cross-sectional studies has shown that SSB consumption is associated with a higher risk of MetS in adults [15,16], although cross-sectional studies cannot establish causality. Nevertheless, prospective studies showed inconclusive results. For example, a cohort study showed a positive association only in women [17], another only with a high SSB consumption [16], and some prospective studies showed no association [18,19]. 

The currently available evidence on the association of SSB consumption and MetS includes three previous meta-analyses. The first one was performed by Malik et al. in 2010 [20]. It has been twelve years since this publication, and an update is needed as new scientific evidence has been produced. On the other hand, the meta-analysis by Narain et al. [21] conducted in 2016 omitted relevant articles. Finally, the meta-analysis by Zhang et al. performed in 2020 [22] included some studies in which the independent association of SSBs could not be separated. For example, the results coming from dietary patterns, as well as from total sweetened beverages (comprising both SSBs as well as ASBs), were included in this meta-analysis. Finally, the distinction between cross-sectional and prospective analysis was not made, giving us a lesser likelihood of suggesting cause-effect relationships. It is important to differentiate studies with different epidemiological designs since in cross-sectional designs causality cannot be associated, while the longitudinal designs help us to suggest cause-effect relationships. Mixing these two types of design in an analysis can alter the results obtained and modify the effect obtained in the long term by longitudinal studies.

Therefore, the aim of the present study was to update and summarize the current information on the association between the consumption of SSBs (soft drinks, bottled fruit juices, energy drinks, and milkshakes), and MetS in adults by performing a meta-analysis that includes the newly available evidence, avoids studies that were misclassified and shows results according to their study design. 

## 2. Materials and Methods

### 2.1. Data Sources and SEARCHES

This meta-analysis followed the Cochrane Handbook for Systematic Reviews of Intervention [23], and was conducted according to the PRISMA statement recommendations. Results were reported according to the Meta-analysis of Observational Studies in Epidemiology (MOOSE) [24] and Preferred Reporting Items for Systematic Reviews and Meta-analyses (PRISMA) reporting guidelines [25]. 

A review of observational studies was conducted to assess the association between the consumption of SSBs and the MetS. Data sources included Pubmed and SCOPUS from database inception to June 2022 (included). In addition, a secondary manual search was conducted, including articles from bibliographic references. Search terms reflected the main sources of SSBs, the outcome of interest, as well as a limitation for languages (Table 1). 

### 2.2. Study Selection

Inclusion criteria were defined based on the following aspects: (a) studies assessing the SSBs-MetS, soft drinks-MetS, or bottled fruit juices-MetS, or energy drinks-MetS, or milkshakes-MetS relationships in population-based epidemiological studies (cross-sectional or longitudinal studies) and conducted in human adults; (b) studies reported Hazard Ratios (HR), Relative Risk (RR) or Odds Ratio (OR) with 95% Confidence Intervals (CI); (c) and studies with sufficient information also reported on risk estimates for the MetS according to categories of SSB consumption or when SSB consumption was considered as a continuous variable.

**Figure 1:**
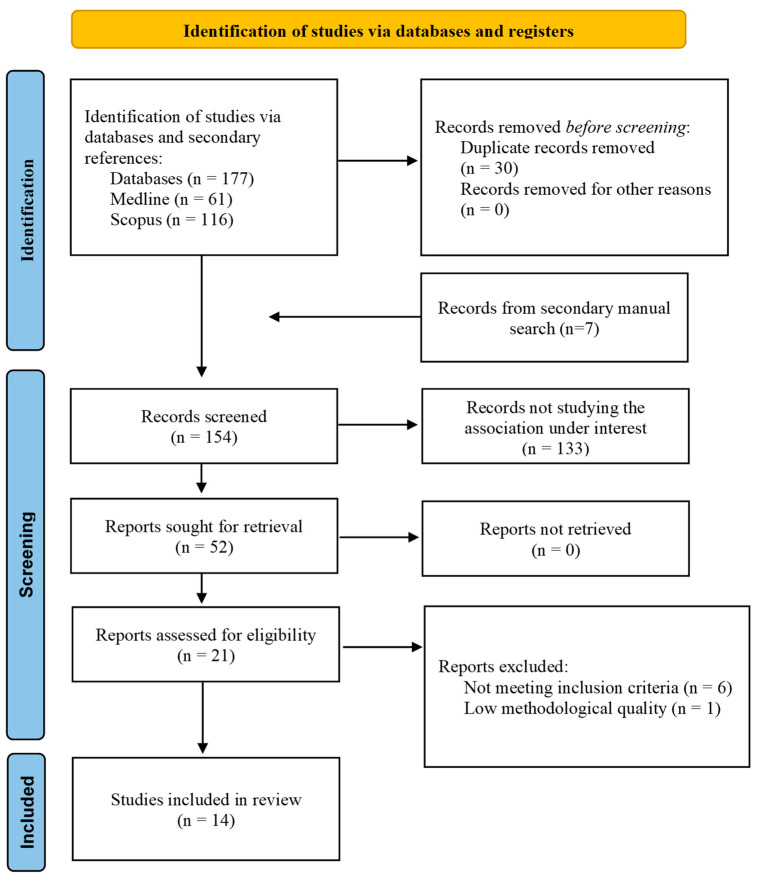
PRISMA 2020 flow diagram for systematic reviews.

Exclusion criteria were defined based on the following: (a) studies conducted in children, adolescents, or pregnant women, due to the fact that in these populations some criteria for the definition of the MetS are not normative, and therefore, the MetS definition could not be comparable; (b) studies assessing the association of the MetS with dietary patterns, or substitution analyses when substituting SSBs or ASBs; (c) studies reporting results on total sweetened beverages, without distinguishing their categories (SSBs and ASBs); (d) studies conducted in selected populations (e.g., on secondary cardiovascular prevention or on patients with kidney disease). (e) studies with low epidemiological quality because the validity of study results is threatened. Additionally, we excluded reviews, meta-analyses, conference articles, and articles for which the full text was not available, or articles with important missing information for the meta-analysis (Figure 1).

### 2.3. Data Extraction and Quality Assessment

Two independent reviewers (AMC and BMF) extracted relevant data from the selected studies, including sample size, participants’ characteristics, exposure measurement (sources of SSBs), dietary assessment, and diagnosis of the MetS, as well as the central estimates for the association (HR, RR, or OR) along with their 95% CIs for the MetS risk when comparing the highest vs. the lowest levels of SSB consumption. When models with different degrees of adjustment were reported, results from the fully adjusted models were selected. 

Quality assessments for the included studies were performed using the Joanna Briggs Institute’s (JBI) critical appraisal checklist for cross-sectional and longitudinal studies as appropriate [26]. We excluded studies rating lower than 7 out of 8 for cross-sectional designs, and lower than 9 out of 11 for cohort studies. 

### 2.4. Statistical Methods/Analysis

We calculated pooled ORs for cross-sectional studies as well as cohort studies. Heterogeneity was assessed by using Cochran’s Q and the I^2^ statistic. A random effects models were used to estimate the pooled ORs with their 95% CI due to the fact that heterogeneity among studies was I^2^ ε 50%. When an article reported data separately for men and women, we introduced the data as independent studies. Publication bias was examined through visual inspection of the funnel plots and by calculating the Egger’s test [27] (*p* values < 0.05 indicate the presence of publication bias). Analyses were performed with Review Manager (version 5.4.1) and R statistical software (version 4.0.4). 

## 3. Results

We identified 14 high-quality articles [nine cross-sectional [15,16,28,29,30,31,32,33,34] and 5 cohort studies [17,18,19,35,36] yielding findings on the following relationships: SSBs-MetS, soft drinks-MetS, or bottled fruit juices-MetS, or energy drinks-MetS, or milkshakes-MetS. More in particular, we found eight articles studying the SSBs-MetS relationship [15,16,19,30,31,34,35,36], four articles studying the soft drinks-MetS relationship [17,29,34,35], two articles assessing bottled fruit juices-MetS relationship [28,36], one article that studied energy drinks-MetS relationship [32], and zero articles assessing the milkshakes-MetS relationship. We excluded the article by Dhingra et al. [37] due to not meeting the quality requirements (Table 2 and Table 3).

Six studies were conducted with data from Asia [15,16,17,28,29,30,31,32,33], three studies from Europe [34,35,36], five studies from America [18,19,30,33,34], and one study was conducted from Oceania [32] and corresponding to eight different countries. All studies analyzed both sexes, but only four studies showed additional sex-specific analysis [15,16,17,29]. Among all the studies, more than half defined the MetS based on National Cholesterol Education Program Adult Treatment Panel III (NCEP ATP III) guidelines [15,16,17,28,29,30,33,34]. After quality assessment, most of the studies rated 8 in the JBI score for cross-sectional studies and 9 for longitudinal studies.

### 3.1. Cross-Sectional Studies Results

**Figure 2:**
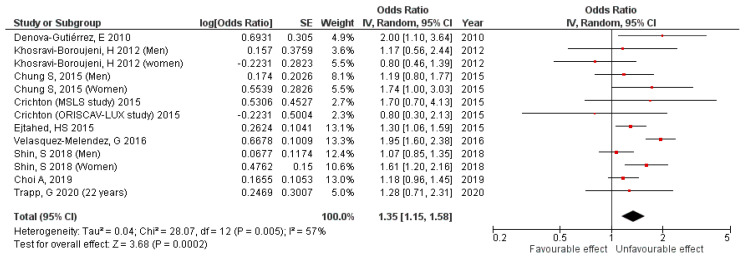
Cross-sectional studies forest plot.

In cross-sectional studies, which included 62,693 adults, the results show a 35% (pooled OR 1.35, 95%CI 1.15,1.58, *p* = 0.0002) increase in the MetS risk for adults with a high SSB consumption, with a moderate heterogeneity among the studies (I^2^ = 57%; *P*_heterogeneity_ = 0.005) (Figure 2). A publication bias was not observed by examination of the funnel plot (Supplementary Figure S1) nor the Egger’s test (*p* = 0.685).

### 3.2. Cohort Studies Results

**Figure 3:**
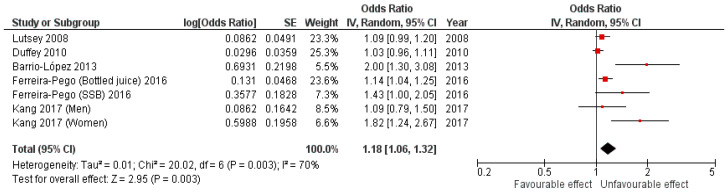
Cohort studies forest plot.

The results for the analysis of cohort studies, which included 28,932 adults, show an 18% (pooled OR 1.18, 95%CI 1.06,1.32, *p* = 0.003) increase in the MetS risk for adults with a high SSB consumption, with a high heterogeneity among the studies (I^2^ = 70%; *P*_heterogeneity_ = 0.003) (Figure 3). We observed publication bias by examination of the funnel plot (Supplementary Figure S2) and Egger’s test (*p =* 0.019). Moreover, among cohort studies, a meta-regression was performed by studying the evolution of the logarithms of the OR versus the year of the publication of the articles. We found that for every one-year increase, the risk of the MetS increased by 2.3%, but without reaching statistical significance (*p* = 0.458).

## 4. Discussion

The findings from our meta-analyses, based on results from the 14 high-quality population-based epidemiological studies and including a total of 91,625 adults, show a positive link between SSB consumption and the risk of the MetS. The results from cross-sectional studies show that the adults in the highest category of consumption had a 35% greater risk of the occurrence of the MetS when compared with those in the lowest category of consumption. The corresponding result for the longitudinal studies was an 18% greater risk of the incidence of the MetS. For the cohort studies, some evidence of publication bias was identified.

Our results are in accordance with the previous meta-analyses that assessed the relationship between SSB consumption and the MetS, despite the existence of methodological differences. The meta-analysis by Malik et al. [20] pooled the results of three prospective cohort studies, in which SSB consumption was associated with a 20% increased risk of developing the MetS (pooled OR 1.20, 95%CI 1.02, 1.42), although some degree of error cannot be ruled out due to the inclusion of ASB consumption. The pooled results from the meta-analysis by Narain et al. [21], in which both children, as well as adults, were included, showed that the cross-sectional analysis suggested a 46% increased risk of the MetS (pooled OR 1.46, 95%CI 1.18, 1.81, *p* = 0.0005). Moreover, three prospective cohort studies were meta-analyzed, but statistical significance was not achieved (pooled OR 1.47, 95%CI 0.89, 2.43, *p* = 0.13). Again, in this meta-analysis, the results from ASB consumption were included. In the meta-analysis by Zhang et al. [22], the results suggested that there was a 56% increased risk of the MetS when the extreme groups of consumption were compared. As the previous one, it was conducted on both children, as well as in adults. They obtained a 19% increased risk for every 250 mL/day of SSBs consumed. In this last meta-analysis, they mixed cross-sectional and cohort studies, and, for some included studies, it was not possible to separate the effects of the consumption of SSBs from ASB consumption.

The positive association between SSB consumption and the risk of the MetS might be explained by multiple potential biological mechanisms. First of all, SSB consumption leads to weight gain, dyslipidemia, as well as insulin resistance due to the high added sugar content and their common elaboration with different varieties of fructose. The extra calories consumed from SSBs are not usually offset by a lower intake of energy from solid food nor with an increment in energy expenditure, in turn, leading to weight gain. SSBs, as forms of liquid carbohydrates, produce less satiety than the equivalent amount of carbohydrates from solid food [38]. Additionally, excessive SSB consumption increases lipogenesis secondary to hepatic fructose metabolism [39,40]. Moreover, fructose is metabolized by the liver, resulting in dyslipidemia [41] and liver-induced hyperuricemia, again leading to insulin resistance as well as an increased risk of the MetS [42,43]. Moreover, the high glycemic load after SSB consumption promotes pro-inflammatory cytokines released in response to hyperglycemia [44].

In our meta-analysis, it is of note that the number of servings was not comparable across studies, so we were only able to compare extreme categories of SSB consumption. Therefore, it is possible that some degree of non-differential misclassification somewhat weakened the pooled estimate. Moreover, there is a substantial variation in study designs and in the exposure assessment across studies, which can explain the large degree of heterogeneity. However, despite the existence of heterogeneity, the central estimates were greater than one in all cohort studies.

The publication bias was explored in our meta-analysis to assess the presence of findings in favor of positive results [45]. In cross-sectional studies, a visual inspection of funnel plots and a standard test suggested no evidence of a publication bias, but we observed a publication bias when the cohort studies were analyzed. However, there is of note that, among cohort studies, four studies have been published with results close to one. Moreover, the low number of articles included could contribute to publication bias, and Egger’s test is less reliable when lower than 10 studies are meta-analyzed [46,47].

All the studies included in our meta-analysis considered adjustments for potential confounding factors, such as sociodemographic, clinical, as well as lifestyle and dietary factors. SSB consumption is usually associated with a higher intake of saturated, trans-saturated fatty acids, daily caloric intake, lower dietary fiber [18], and lower levels of physical activity [30,31]. For most of the studies, a positive association persisted after adjustments, suggesting an independent effect of SSBs on the occurrence of MetS. However, some residual confounding due to an incomplete adjustment could still persist, resulting in an overestimation of the strength of the association. This overestimation could be more relevant among cross-sectional studies, as few studies adjusted their results for BMI nor dietary factors other than energy intake [28,30,31], while all prospective studies took into account dietary confounding factors.

Our study has some strengths. First, to explore the separate association between SSB consumption and MetS risk, not including those studies where ASB consumption could influence the results. Second, to update the evidence through the inclusion of original articles missed in previous meta-analyses. Third, we excluded the studies with incomplete data, misclassifications, or errors in data analysis, as well as studies with a low methodological quality. Fourth, the *Cochrane Handbook for Systematic Reviews of Intervention* and the PRISMA guidelines were followed when performing the meta-analysis as well as when reporting the results. Fifth, we show data on cross-sectional and cohort studies, performing separate analyses for each type of design. Lastly, we provide explanations for the weaker results in prospective studies.

Our study also has some limitations. Most of the included studies were cross-sectional, preventing us from establishing a temporal relationship between SSB consumption and the occurrence of the MetS, although separate analyses, according to their design, attenuated this limitation. Second, our results show a high degree of heterogeneity among studies. This heterogeneity might be related to differences in the exposure measurement, the MetS diagnosis criteria, the length of the follow-up periods, as well as the adjustment for confounders, although the association was positive for all the included cohort studies. Moreover, this meta-analysis is limited by the existing evidence. A scarcity of prospective cohort studies was shown, also resulting in publication bias. Finally, the number of servings was not comparable across studies, probably deriving an underestimation of the association.

## 5. Conclusions

A higher SSB consumption is positively associated with the MetS occurrence. In the future, the publication of more studies assessing the prospective association is desirable. Meanwhile, public health authorities must pay attention in order to implement general public strategies to discourage SSB consumption and promote the prevention of the MetS.

## Figures and Tables

**Table 1:** Search strategy in selected databases.

PubMed	((“sugar sweetened beverages”[MeSH Terms] OR (“sugar sweetened”[All Fields] AND “beverages”[All Fields]) OR “sugar sweetened beverages”[All Fields] OR (“sugar”[All Fields] AND “sweetened”[All Fields] AND “soft”[All Fields] AND “drinks”[All Fields]) OR “sugar sweetened soft drinks”[All Fields] OR (“fruit and vegetable juices”[MeSH Terms] OR (“fruit”[All Fields] AND “vegetable”[All Fields] AND “juices”[All Fields]) OR “fruit and vegetable juices”[All Fields] OR (“fruit”[All Fields] AND “juices”[All Fields]) OR “fruit juices”[All Fields]) OR (“energy drinks”[MeSH Terms] OR (“energy”[All Fields] AND “drinks”[All Fields]) OR “energy drinks”[All Fields]) OR (“milkshake”[All Fields] OR “milkshakes”[All Fields])) AND (“metabolic syndrome”[MeSH Terms]) AND ((“english”[Language] OR “spanish”[Language]) AND “adult”[MeSH Terms])) AND ((english[Filter] OR spanish[Filter]) AND (alladult[Filter])).
SCOPUS	TITLE-ABS-KEY (“sugar sweetened soft drinks” OR “fruit juices” OR “energy drinks” OR “milkshakes”) OR INDEXTERMS (“sugar sweetened beverages” OR “fruit and vegetable juices” OR “energy drinks”) AND INDEXTERMS (“metabolic syndrome”) AND (LIMIT-TO (SRCTYPE, “j”)) AND (LIMIT-TO (DOCTYPE, “ar”)) AND (LIMIT-TO (LANGUAGE, “English”) OR LIMIT-TO (LANGUAGE, “Spanish”)).

**Table 2:** Characteristics of the cross-sectional studies sorted by inclusion status and chronological year of publication.

Author (Year)	Country	Age Range (y)	Sex	Characteristics of Subjects	Sample Size	Exposure	Exposure Categories	Dietary Assessment	Diagnosis Criteria for the Metabolic Syndrome (Number of Events)	OR (95%CI) for Highest vs. Lowest Intake	Adjustment for Confounders	Quality Score (JBI Criteria Not Met)
Denova-Gutiérrez et al. (2010) [30]	Mexico	20–70 y	M-W	Participants from the Health Workers Cohort Study in the Mexican states of Morelos and Mexico	5240 participants (1488 men and 3752 women)	SSB: colas, flavored sodas, flavored water with sugar and diet colas	0 servings/day,1 serving/day, 1–2 servings/day, 2 servings/day	FFQ.	NCEP ATP III (cut-off for plasma glucose level of ≥5.6 mmol/L)	2.0 (1.10, 3.64) *p* value (not shown)	Age, sex, BMI, weight change within past year, physical activity, energy intake, alcohol intake, SFA intake, PUFA intake, trans fatty acid intake, smoking, and place of residence	8/8 Included
Khosravi-Boroujeni et al. (2012) [31]	Iran	>19 y	M-W (stratified)	Participants from the Isfahan Healthy Heart Program (IHHP)	1752 participants (782 men and 970 women)	SSB: soft drinks plus artificially sweetened fruit juices	<1 time/week, 1–3 times/week, ≥times/week	FFQ	ATP III.	SSB: Men: 1.17 (0.56–2.44) *p* = 0.57 SSB: Women: 0.80 (0.46–1.39) *p* = 0.59	Age, BMI, smoking, physical activity, total energy intake, dietary intake of meat, grains, pulses, fruit, vegetable, dairy, HVOs, and non-HVOs	8/8 Included
Chung et al. (2015) [29]	South Korea	≥30 y	M-F (stratified)	Participants from the 2007–2011 Korea National Health and Nutrition Examination Survey (KNHANES)	13,972 participants (5432 men, and 8540 women)	Soft drinks	Rarely, ≤1 time/month, 2–3 times/month, 1 time/week, 2–3 times/week, ≥4 times/week	Dietary questionnaire and 24-h dietary recall	NCEP ATP III, [waist circumference (WHO ethnicity-specific cut-off values for the Asian population) ≥90 cm for men and 80 cm for women]	Men: 1.19 (0.80–1.77), *p* = 0.7890 Women: 1.74 (1.00–3.03), *p* < 0.0001	Age, sex, family income, education, current smoking status, physical activity total, energy intake, and alcohol intake	8/8 Included
Crichton et al. (2015) [34]	USA and Luxemburg	23–98 y (MSLS), 18–69 y (ORISCAV-LUX)	M-W	Participants from MSLS study and ORISCAV-LUX study	2126 participants (803 from MSLS and 1323 from ORISCAV-LUX)	Soft drinks	Non-consumers, one per day, two or more per day	FFQ	NCEP ATP III. (*n* in MSLS = 353) (*n* in ORISCAV-LUX = 346)	MSLS: 1.7 (0.7–4.5), *p* > 0.05 ORISCAV-LUX: 0.8 (0.3–1.8), *p* = 0.05	Age, sex, education, smoking, physical activity, total energy intake, alcohol intake, intake of vegetables, fruit, grains, meat, and diet soft drinks	8/8 Included
Ejtahed et al. (2015) [15]	Iran	19–70 y	M-W	Participants from the fourth phase of TLGS (from 2009 to 2011)	5852 participants (2516 men and 3336 women)	SSB: soft drinks plus and bottle fruit juices	Using quartile cutoffs (<6.7, from 6.7 to 21.8, from 21.9 to 57.1, >57.1 g/day). Participants with dietary SSB intakes <6.7 g/day were considered as the reference group	FFQ	NCEP ATP III	1.3 (1.06–1.59) *p* = 0.03	Age, sex, education, smoking, physical activity, and total energy intake	8/8 Included
Velasquez-Melendez et al. (2016) [33]	Brazil	35–74 y	M-W	Participants from the ELSA-Brasil study	8826 participants (3950 men, and 4876 women)	Soft drinks	<0.1 serving/day, 0.1 to <0.4 serving/day, 0.4 to <1 serving/day, and ≥1 serving/day	Beverage frequency questionnaire	NCEP ATP III. (*n* = 1314)	1.95 (1.60–2.38) *p* < 0.001	Age, sex, income, education, smoking, physical activity, energy intake, alcohol intake, and daily consumption of fruit and vegetables	8/8 Included
Shin et al. (2018) [16]	South Korea	35–65 y	M-W (stratified)	Participants from the 2012–2016 KNHANES.	12,112 participants (5308 men, and 6804 women)	SSB: soda beverages, fruit juices and sweetened rice drinks	Non-SSB drinkers, ≤2 times/week, 3–6 times/week, and ≥1 times/day	FFQ	NCEP ATP III, [waist circumference (WHO ethnicity-specific cut-off values for the Asian population) ≥90 cm for men and 80 cm for women] (*n* in men = 1717) (*n* in women = 1518)	Men: 1.07 (0.85–1.35) *p* = 0.0989 Women: 1.61 (1.20–2.16) *p* = 0.0003	Age, family income, educational, energy intake, alcohol intake, smoking status, and physical activity	8/8 Included
Choi et al. (2019) [28]	South Korea	19–74 y	M-W	Participants from the KNHANES study	10,460 participants (4082 men and 6378 women)	Fruit juices.	Rarely, from 1 to 3 times/month, and ≥1 time/week	FFQ	NCEP ATP III, [waist circumference (World Health Organization ethnicity-specific cut-off values for the Asian population) ≥90 cm for men and 80 cm for women]	1.18 (0.96–1.45) *p* = 0.1161	Age, sex, family income, education, BMI, smoking, physical activity, total energy intake, alcohol intake, sugar intake from processed food, dietary pattern 1, and dietary pattern 2	8/8 Included
Trapp et al. (2020) [32]	Australia	20 y and 22 y	M-W	Participants from the Raine Study Generation 2	2353 participants (1236 of 20 y, and 1117 of 22 y)	Energy drinks	none/rare (never to ≤once/month); occasional (>once/month to <once/week); frequent (≥once/week)	Self-reported questionnaire	International Diabetes Foundation (*n* after 20 y = 73) (*n* after 22 y = 92)	20 y: 1.11 0.57–2.19), *p* > 0.05 22 y: 1.28 (0.71–2.31), *p* > 0.05	Sex, family income, mother’s education, education, smoking, physical activity, energy intake, alcohol intake, and dietary pattern	7/8 (JBI: 2) Included
Dhingra et al. (2007) [37]	USA	Adults	M-W	Participants from the Framingham Offspring Study	8997 participants (4126 men and 4871 women)	Soft drinks.	From 1 to 6 soft drink/week, ≥1 soft drink/day	FFQ.	NCEP ATP III. (*n* = 2777)	1.81 (1.28–2.56)	Age, sex, physical activity, smoking, energy intake, dietary intake of SFA, trans fat, fiber, magnesium, and glycemic index	5/8 (JBI: 3, 4, 8) Excluded

BMI: body mass index; CI: confidence interval; FFQ: food frequency questionnaire; HVOs: hydrogenated vegetable oil; JBI: Joanna Briggs Institute; M-W: men-women; NCEP ATP III: National Cholesterol Education Program Adult Treatment Panel III; OR: odds ratio; PUFA: polyunsaturated fatty acids; SFA: saturated fatty acids; SSB: sugar-sweetened beverage. JBI criteria for analytical cross-sectional studies: (1) criteria for inclusion; (2) detailed description of the study subjects; (3) exposure measurement; (4) standard criteria used for exposure measurement; (5) confounding factors; (6) strategies to deal with confounders; (7) outcome measurement; (8) statistical analysis.

**Table 3:** Characteristics of the cohort studies sorted by inclusion status and chronological year of publication.

Author (Year)	Country	Age Range (y)	Sex	Characteristics of Subjects	Sample Size	Follow-Up	Exposure	Exposure Categories	Dietary Assessment	Diagnosis Criteria for the Metabolic Syndrome (Number of Events)	OR (95%CI) for Highest vs. Lowest Intake	Adjustment for Confounders	Quality Score (JBI Criteria Not Met)
Lutsey et al. (2008) [18]	USA	45–64 y	M-W	Participants from ARIC study	9514 participants (4197 men and 5317 women)	9-year-follow-up	SSBs	Tertiles of beverage consumption (T1 considered as reference)	FFQ.	American Heart Association guidelines (*n* = 3782)	1.09 (0.99–1.19), *p* = 0.07	Age, sex, center, race, education, smoking, physical activity, energy intake, consumption of meat, dairy, fruit and vegetables, whole grains, and refined grains	9/11 (JBI: 9, 10) Included
Duffey et al. (2010) [19]	USA	18–30 y	M-W	Participants from de Coronary Artery Risk Development in Young Adults (CARDIA) study	3596 participants.	Data were used from exam years 0 (1985–1986, baseline), 7 (1992–1993), and 20 (2005–2006)	SSBs	Quartiles of beverage consumption (average of years 0 and 7)	FFQ.	ATP III. (*n* = 459)	1.03 (0.96, 1.11), *p* = 0.401	Age, sex, CARDIA center race, weight, smoking, physical activity, energy intake, alcohol intake, energy from low-fat milk, whole-fat milk, and fruit juices	9/11 (JBI: 9, 10) Included
Barrio-Lopez et al. (2013) [34]	Spain	>18	M-W	Participants from The Seguimiento Universidad de Navarra (SUN) Project	8157 participants.	6-year-follow-up	SSBs: sugar-sweetened carbonated colas and fruit-flavored carbonated sugar soft drinks	Quintiles of change in beverage consumption (quintile 1 for those participants who decreased most of their consumption and quintile 5 for those participants who increased most of their consumption), considering the first quintile as the reference category	FFQ.	The International Diabetes Federation, the American Heart Association, and National Heart, Lung, and Blood Institute (*n* = 361)	2.0 (1.30, 3.08), *p* = 0.038	Age, sex, BMI, smoking, physical activity, energy intake, alcohol intake, soft drink consumption, consumption of red meat, French fries, fast food, and adherence to the Mediterranean dietary pattern	10/11 (JBI: 10) Included
Ferreira-Pêgo et al. (2016) [36]	Spain	Men aged 55–80 y, and women aged 60–80 y	M-W	Patients from the PREDIMED study.	1868 participants	October 2003 to June 2009	SSBs and bottled fruit juices	<1 serving/week, 1–5 servings/week, >5 servings/week.	FFQ	The International Diabetes Federation, the American Heart Association, and National Heart, Lung, and Blood Institute (*n* for SSBs = 936) (*n* for bottled fruit juices = 944)	SSBs: 1.43 (1.00, 2.05), *p* = 0.27 Bottled fruit juices: 1.14 (1.04, 1.25), *p* = 0.31	Age, sex, intervention group, BMI, smoking, physical activity, cumulative energy intake, alcohol intake, alcohol squared in grams per day, cumulative mean consumption of vegetables, legumes, fruit, cereals, meat, fish, bakery, dairy products, olive oil, and nuts, and MetS components at baseline	9/11 (JBI: 9, 10) Included
Kang et al. (2017) [17]	South Korea	50–69 y	M-W (stratified)	Participants from KoGES cohort study	5797 participants (3027 men and 2770 women)	10-year-follow-up	Soft drinks	none or rarely, <1 serving/week, ≥1 serving/week to <4 servings/week and ≥4 servings/week	FFQ	NCEP ATP III. (*n* in men = 1046) (*n* in women =1083)	Men: 1.09 (0.79, 1.50), *p* = 0.9531 Women: 1.82 (1.24, 2.67), *p* < 0.001	Age, income, education, BMI, smoking physical activity, energy intake, alcohol intake, percentage of fat, fiber intake, and the presence of diseases	9/11 (JBI: 9, 10) Included
Dhingra et al. (2007) [37]	USA	Adults	M-W	Participants from Framingham Offspring Study from the fourth through the seventh (1998–2001) examination cycles	6039 participants (2569 men and 3470 women)	4-year-follow-up	Soft drinks	From 1 to 6 soft drink/week, ≥1 soft drink/day	FFQ.	NCEP ATP III. (*n* = 1150)	1.29 (0.98–1.70) *p* value (not shown)	Age, sex, smoking, physical activity, energy intake, dietary intake of SFA, trans fat, fiber, magnesium, and glycemic index	7/11 (JBI: 2, 3 9, 10) Excluded

BMI: body mass index; CI: confidence interval; FFQ: food frequency questionnaire; JBI: Joanna Briggs Institute; M-W: men-women; NCEP ATP III: National Cholesterol Education Program Adult Treatment Panel III; OR: odds ratio; SFA: saturated fatty acids; SSB: sugar-sweetened beverage. JBI criteria for cohort studies: (1) similar groups and from the same population; (2) exposure measured similarly in exposed and unexposed groups; (3) exposure measurement; (4) confounding factors; (5) strategies to deal with confounders; (6) free of the outcome at the start of the study; (7) outcome measurement; (8) follow-up time reported and sufficient; (9) losses to follow-up; (10) strategies to address incomplete follow-up; (11) statistical analysis.

## Data Availability

Data described in the manuscript, code book, and analytic code will be made available upon request pending on request from the corresponding author.

## Supplementary Materials

The following supporting information can be downloaded at: https://www.mdpi.com/article/10.3390/nu15020430/s1, Figure S1: Cross-sectional studies funnel plot; Figure S2: Cohort studies funnel plot.
