# Supplementary figures and images for: Association between Sugar-Sweetened Beverage Consumption and the Risk of the Metabolic Syndrome: A Systematic Review and Meta-Analysis
Source: Nutrients. 2023 Jan 13;15(2):430. doi: 10.3390/nu15020430 (PMC9912256; doi:10.3390/nu15020430)

**Supplemental Figure S1.** Cross-sectional studies funnel plot.

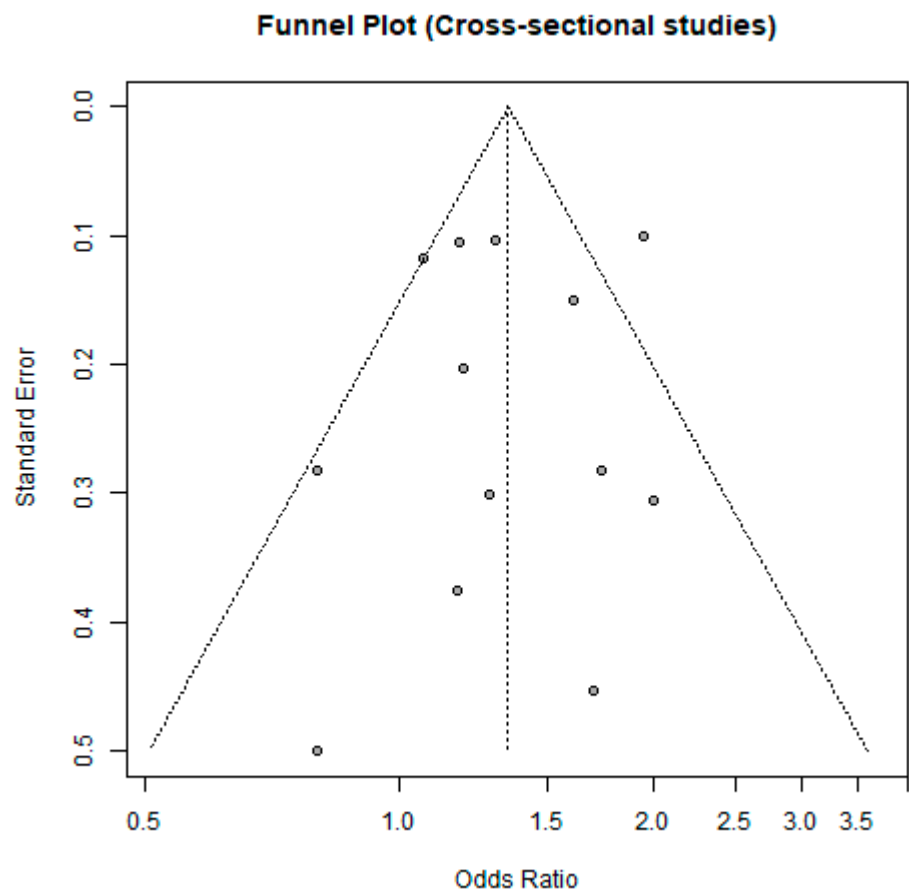

Supplement: Supplementary file 1 [file nutrients-15-00430-s001.zip › Supplemental Figure S1_Cross-sectional studies funnel plot.pdf]

**Supplemental Figure S2.** Cohort studies funnel plot.

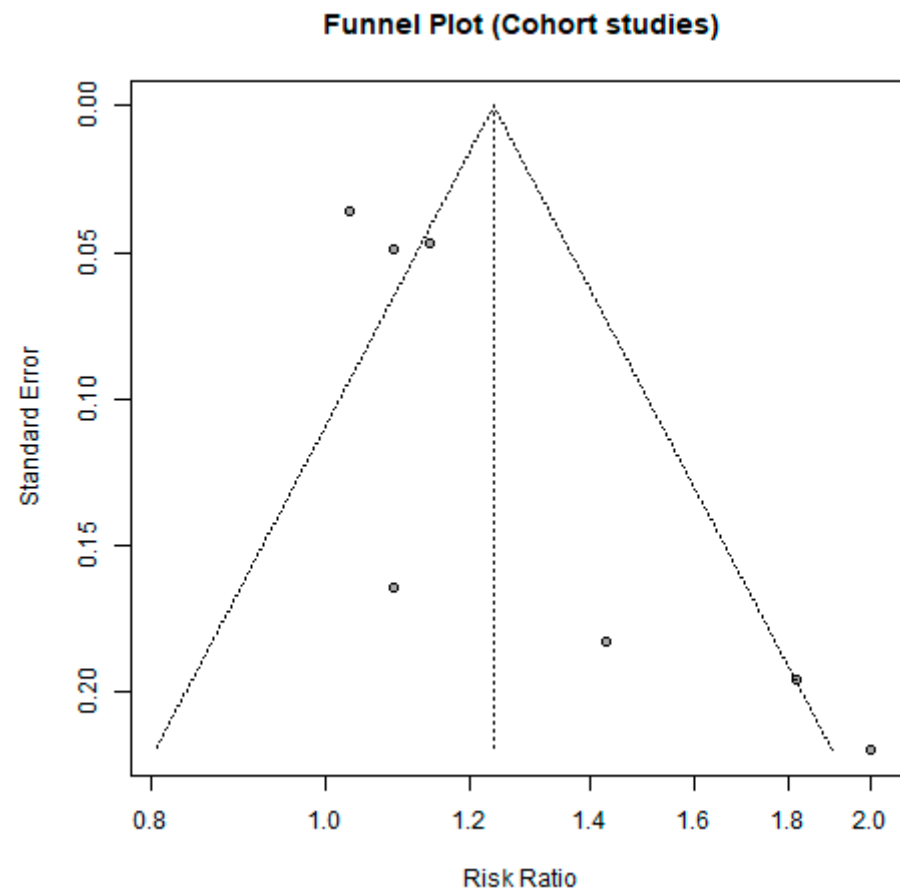

Supplement: Supplementary file 1 [file nutrients-15-00430-s001.zip › Supplemental Figure S2_Cohort studies funnel plot.pdf]
